# 肺癌患者入院时下肢深静脉血栓的发生率及相关危险因素分析

**DOI:** 10.3779/j.issn.1009-3419.2018.10.05

**Published:** 2018-10-20

**Authors:** 晖 杜, 洪林 赵, 梅 李, 慧慧 纪, 凡 任, 攀 王, 昕 李, 明 董, DAWAR Rehman, 钢 陈, 军 陈

**Affiliations:** 300052 天津，天津医科大学总医院肺部肿瘤外科 Department of Lung Cancer Surgery, Tianjin Medical University General Hospital, Tianjin 300052, China

**Keywords:** 肺肿瘤, 下肢深静脉血栓, 发生率, 危险因素, Lung neoplasms, Lower extremity deep venous thrombosis, Incidence, Risk factors

## Abstract

**背景与目的:**

静脉血栓栓塞症（venous thromboembolism, VTE）是一种公认的在肺癌患者中有较高发病率和死亡率的并发症。本研究目的是明确我们中心肺癌患者入院时下肢深静脉血栓（lower extremity venous thrombosis, LEDVT）的发生率，并揭示其入院时LEDVT发生的危险因素。

**方法:**

选择天津医科大学总医院肺部肿瘤外科在2017年7月-2017年12月收治的231例肺癌患者，入院时即行双下肢静脉彩超检查，以分析肺癌患者LEDVT的发生率；同时，对肺良性疾病患者入院时LEDVT的发生率进行比较。在肺癌患者中，进一步分析LEDVT发生与其临床特征的关系，寻找LEDVT发生的可能危险因素；同时，亦分析这些患者的血浆D-二聚体（D-Dimmer）、纤维蛋白原（fibrinogen, FIB）、凝血酶时间（thrombin time, TT）、活化部分凝血酶时间（activated partial thrombin time, APTT）、凝血酶原时间（prothrombin time, PT）及血小板（platelet, PLT）之间的差异。

**结果:**

在231例肺癌患者中，入院时发生LEDVT者12例，其发生率为5.2%（12/231）；而77例肺良性疾病患者入院时均未查见LEDVT的发生，提示肺癌患者入院时LEDVT的发生率明显高于肺良性疾病（*P* < 0.05）。在肺癌患者中进一步分析发现，伴有远处转移（包括N3淋巴结转移）的肺癌患者较不伴有转移者更易发生LEDVT（11.29%, 7/62 *vs* 2.96%, 5/169）（*P* < 0.05）。肺癌LEDVT组患者入院时的D-Dimer的中位值为1, 534mg/L（369 mg/L-10, 000 mg/L），明显高于非LEDVT组患者(539 mg/L, 126 mg/L-1, 000 mg/L)（*P* < 0.05）；而FIB、TT、APTT、PT和PLT在两组患者之间无明显统计学差异（*P* > 0.05）。

**结论:**

我们中心肺癌患者入院时LEDVT总体发生率约为5%，明显高于肺良性疾病患者。入院时伴有远处转移（包括N3淋巴结转移）的肺癌患者更易发生LEDVT，其中，D-Dimer值较高的患者应考虑到VTE事件发生的可能。

## Abstract (en)

**Background and objective:**

Venous thromboembolism (VTE) is a recognized complication in lung cancer patients with higher morbidity and mortality. The purpose of this study is to determine the incidence of lower extremity venous thrombosis (LEDVT) in lung cancer patients and to reveal the risk factors for LEDVT during admission in our center.

**Methods:**

We first connected 231 patients with lung cancer admitted to the Department of Lung Cancer Surgery, Tianjin Medical University General Hospital from July 2017 to December 2017. All these patients underwent color ultrasound examination of lower extremity vein on admission to analyze the incidence of LEDVT. At the same time, the incidence of LEDVT in patients with benign lung diseases on admission was used as control. In order to explore the possible risk factors for LEDVT in these patients with lung cancer, we further analyze the correlations between LEDVT and their clinical features. At the same time, we also analyze the relationship between LEDVT and Plasma D-Dimmer, fibrinogen (FIB), thrombin time (TT), activated partial thrombin time (APTT), prothrombin time (PT) and platelet (PLT) in these patients with lung cancer.

**Results:**

Among 231 patients with lung cancer, the incidence rate of LEDVT on admission was 5.2% (12/231), and in 77 patients with benign lung disease, there was none of patients with LEDVT on admission. This result indicated that the admitted incidence rate of LEDVT in patients with lung cancer was significantly higher than that in patients with benign lung disease (*P* < 0.05). Further analysis in patients with lung cancer found that there was higher incidence rate of LEDVT in distant metastasis group (including N3 lymph node metastasis) compared to in non-distant metastasis group (11.29%, 7/62 *vs* 2.96%, 5/169) (*P* < 0.05). In patients with lung cancer, the median value of D-Dimer in LEDVT group was 1, 534 mg/L (369 mg/L-10, 000 mg/L), which was significantly higher than that in the non-LEDVT group (539 mg/L, 126 mg/L-1, 000 mg/L) (*P* < 0.05). There was no statistically significant difference in FIB, TT, APTT, PT and PLT between these two groups (*P* > 0.05).

**Conclusion:**

The overall incidence of LEDVT in our central lung cancer patients was approximately 5%, significantly higher than that in patients with benign lung disease. Lung cancer patients with distant metastasis (including N3 lymph node metastasis) at admission were more likely to develop LEDVT, and these patients with higher D-Dimer values should be considered the possibility of VTE events.

目前，肺癌在国内恶性肿瘤中的发病率和死亡率均居首位，已成为当前研究的主要热点问题^[1]^。静脉血栓栓塞症（venous thromboembolism, VTE）包括肺动脉栓塞（pulmonary thromboembolism, PE）和深静脉血栓（deep venous thrombosis, DVT），是一种公认的肺癌并发症，在肺癌患者中有极高的发病率和死亡率^[2, 3]^。据估计，有4%-20%的癌症患者经历过VTE^[4, 5]^。引起肺癌患者发生VTE的原因除肿瘤自身因素、患者自身因素外，还包括肺癌的治疗，如手术、化疗药物、靶向药物、抗血管生成药物等。肿瘤的自身因素及治疗会引起凝血、纤溶系统紊乱、血管内皮细胞破坏、血液动力学改变，从而增加VTE的发病风险^[6]^。肺癌患者的VTE事件会对其治疗产生严重后果，如化疗延迟、出血风险、药物的相互作用、反复的血栓发生、生活质量的下降以及医疗资源的过度消耗等^[7, 8]^。有报道^[9]^称，伴有VTE发生的癌症更具有侵袭性。

DVT主要发生在下肢，称为下肢深静脉血栓（lower extremity venous thrombosis, LEDVT），LEDVT包括下肢近端DVT和小腿DVT，小腿DVT又分为胫腓静脉血栓和小腿肌间静脉血栓（calf muscle venous thrombosis, CMVT）^[10, 11]^。LEDVT是肺癌患者常见并发症之一，若其栓子脱落进一步形成PE，则会对肺癌患者产生严重不良影响。然而，LEDVT的发生具有隐匿性，且其临床表现多无特异性，目前在国内对其研究较少。因此，本研究将对本中心一段时间内的符合纳入标准的肺部肿物患者进行双下肢静脉彩超检查，LEDVT发生率的检测和临床资料的分析，旨在明确本中心肺癌患者入院时LEDVT的发生率，并揭示其发生的相关危险因素。

## 1 资料与方法

### 1.1 临床资料

收集天津医科大学总医院肺部肿瘤外科在2017年7月-2017年12月之间符合纳入标准的308例肺部肿物患者（病理证实，肺癌患者231例，肺部良性疾病患者77例），入院时即行双下肢静脉彩超检查，对其LEDVT的发生率进行统计分析；比较肺癌患者与肺良性疾病患者入院时LEDVT发生率的差异。进一步将肺癌患者分为LEDVT组和非LEDVT组，对两组患者的临床病例资料进行统计分析，如性别、年龄、体质量指数（body mass index, BMI）、高血压史、吸烟史、肿瘤类型、肿瘤远处转移等，探讨肺癌患者入院时LEDVT发生的危险因素。检测肺癌患者入院时的凝血指标，如D-Dimer、FIB、TT、APTT、PT和PLT，以研究其对LEDVT发生患者的检测价值。该研究均获得天津医科大学总医院临床试验伦理委员会的批准和获得患者的知情同意。

### 1.2 纳入标准与排除标准

纳入标准：（1）患者病理明确诊断为肺癌，且入院时未进行过肺癌手术治疗；肺癌转移情况依据手术大病理或者影像学。肺良性疾病均是手术切除后病理诊断；（2）入院时均行双下肢静脉彩超检查和凝血功能、血小板检查。排除标准：（1）患者既往有下肢深静脉血栓病史；（2）入院前口服抗凝药或者抗血小板药的患者；（3）临床住院资料不完整的患者。

### 1.3 统计学方法

数据统计采用SPSS 20.0软件进行统计，计量资料采用*t*检验，检验前检查正态分布和方差齐性，不符合正态分布则采用秩和检验；计数资料采用χ^2^检验；*P* < 0.05为差异具有统计学意义。

## 2 结果

### 2.1 肺癌患者入院时LEDVT的发生率及相关危险因素分析

收集天津医科大学总医院肺部肿瘤外科在2017年7月-2017年12月之间符合纳入标准的308例肺部肿物患者。其中，病理证实肺癌患者231例，肺部良性疾病患者（包括肺结核病、肺炎性假瘤、肺脓肿及肺错构瘤等）77例，入院时即行双下肢静脉彩超检查，对其LEDVT的发生率进行了统计分析。在231例肺癌患者中，入院时行双下肢静脉彩超检查证实，有12例患者存在LEDVT，LEDVT的发生率为5.2%（12/231）；而在最终手术病理证实为肺部良性疾病的77例患者中，入院时双下肢静脉彩超提示0例患者存在LEDVT（0/77）。如表 1所示，CMH-χ^2^检验分析肺癌及肺良性疾病患者入院时LEDVT的发生情况证实，肺癌患者入院时LEDVT的发生率明显高于肺良性疾病患者（*P*=0.041）。

**1:** 肺癌与肺良性疾病患者LEDVT发生情况的比较 Comparison of LEDVT in patients with lung cancer and benign lung diseases

Occurrence of LEDVT	Lung cancer group	Benign lung diseases group	*P*
Yes	12	0	0.041
No	219	77	
LEDVT: lower extremity venous thrombosis.			

进一步将231例肺癌患者分为血栓组和非血栓组，对两组患者的临床特征计数数据进行χ^2^检验分析，包括患者的性别、年龄、BMI、吸烟史、糖尿病史、高血压史、肺癌位置、肺癌组织类型、入院前有无肺癌化疗治疗以及远处转移情况（包括肺癌淋巴结N3转移）。如表 2所示，CMH-χ^2^检验分析发现：入院时伴远处转移（包括肺癌淋巴结N3转移）的肺癌患者LEDVT的发生率为11.29%（7/62），明显高于不伴有远处转移的肺癌患者（2.96%, 5/169）（*P*=0.011）；而入院时肺癌患者的血栓发生率与患者的性别、年龄、BMI、吸烟史、糖尿病史、高血压史、肺癌位置、肺癌组织类型和有无化疗史等无明显相关性（*P* > 0.05）。其中，19例患者在入院前行化疗治疗，但化疗周期均不大于4次（3例患者仅化疗1次，13例患者化疗2次，1例患者化疗3次，2例患者化疗4次）。

**2:** 231例肺癌患者血栓组与非血栓组的临床资料分析 Clinical analysis of 231 patients with lung cancer in LEDVT group and non-LEDVT group

Clinical data		LEDVT group	Non-LEDVT group	*P*
Gender	Male	9	135	0.353
	Female	3	84	
Age (yr)	> 60	8	142	0.897
	≤60	4	88	
BMI (kg/m^2^)	≥25	3	75	0.510
	< 25	9	144	
Somking history	No	4	98	0.438
	Yes	8	121	
Diabetes history	No	1	22	0.847
	Yes	11	197	
Hypertension history	No	9	145	0.529
	Yes	3	74	
Tumor location	Left	6	92	0.586
	Right	6	127	
Chemotherapy	No	10	202	0.274
	Yes	2	17	
Pathological type	Adenocarcinoma	8	122	0.690
	Squamous carcinoma	3	61	
	Other	1	36	
Distant metastasis (including N3)	No	5	164	0.011
	Yes	7	55	

如表 3所示，12例VTE的肺癌患者中，5例为左侧CMVT，且其中1例合并左侧胫腓静脉血栓；2例为孤立性右侧CMVT；3例为双侧CMVT，其中1例合并下肢近端DVT；3例患者存在下肢近端DVT，其中2例患者的CTPA提示存在双肺动脉栓塞。

**3:** 12例肺癌患者VTE的发生情况及其D-Dimer值 Occurrence VTE of 12 cases of lung cancer and its D-Dimer value

Order number	Occurrence of VTE	D-Dimer value
1	Bilateral CMVT, bilateral popliteal vein thrombosis and distal femoral vein thrombosis	7, 907
2	Left popliteal vein thrombosis, bilateral superficial femoral veinthrombosis, bilateral PE	10, 000
3	Bilateral popliteal vein thrombosis with bilateral PE	9, 349
4	Bilatera CMVT	2, 659
5	Bilatera CMVT	1, 422
6	Left CMVT and left tibiofibular vein thrombosis	1, 776
7	Left CMVT	527
8	Left CMVT	1, 560
9	Left CMVT	1, 508
10	Left CMVT	885
11	Right CMVT	369
12	Right CMVT	449
VTE: venous thromboembolism; CMVT: calf muscle venous thrombosis; PE: pulmonary thromboembolism.		

### 2.2 肺癌LEDVT组和非LEDVT组患者入院时凝血指标的分析比较

进一步对肺癌LEDVT组和非LEDVT组患者入院时的D-Dimer、FIB、TT、APTT、PT和PLT数据进行收集和分析。由于患者的D-Dimer、FIB、APTT、PT及血小板的数据不符合正态性，因此采用最小值、最大值及中位值来描述数据，并进行秩和检验；而患者的TT数据符合正态性，因此采用均数±标准差（Mean±SD）来描述数据，并进行*t*检验。结果如表 4所示，肺癌LEDVT组患者入院时的D-Dimer的中位值为1, 534 mg/L（369 mg/L-10, 000 mg/L），明显高于非LEDVT组患者（539 mg/L, 126 mg/L-1, 000 mg/L）（*P*=0.001）；而FIB、TT、APTT、PT和PLT在两组患者之间无明显统计学差异（*P* > 0.05）。而在12例VTE患者中，伴有PE或者下肢近端DVT患者的D-Dimer平均值为远高于CMVT患者（表 4）。

**4:** LEDVT组与非LEDVT组患者凝血指标的分析 Analysis of coagulation indexes in lung cancer patients with LEDVT and non-LEDVT group

Coagulation index	LEDVT group (*n*=12)	Non-LEDVT group (*n*=219)	*P*
D-Dimer (mg/L)	369-10, 000 (1, 534)	126-1, 000 (539)	0.001
FIB (g/L)	5.96-1.65 (3.94)	2.16-8.4 (3.67)	0.890
TT (Mean±SD, s)	19.36±2.13	18.71±1.85	0.245
APTT (s)	23-38.8 (28.05)	16.6-43.8 (29.5)	0.086
PT (s)	10.4-12.5 (11.35)	9.1-19.4 (11.0)	0.189
PLT (×10^9^/L)	129-405 (243)	88-695 (248)	0.896
FIB: fibrinogen; TT: thrombin time; PLT: platelet; APTT: activated partial thrombin time; PT: prothrombin time.				

## 3 讨论

肺癌是目前国内发病率和死亡率最高的恶性肿瘤，已成为当前研究的热点问题^[1]^。由于目前微创外科的普遍使用，肺癌患者术后并发症有所减少，但是下肢静脉血栓（lower extremity venous thrombosis, LEVT）包括LEDVT、下肢浅静脉血栓（lower extremity superficial venous thrombosis, LESVT）和CMVT，仍是肺癌患者最常见的并发症之一。肺癌患者的LEVT事件会对其治疗产生严重后果，如出血风险、化疗延迟、血栓复发、生活质量的下降以及医疗资源的过度消耗等^[12, 13]^。有报道称，与血栓形成相关的恶性肿瘤更具有侵袭性，且预后更差^[14]^；然而，在国内虽然有不少针对肿瘤患者伴发VTE的研究，但是单独对肺癌患者LEVT发生的相应研究却较少。本文就肺癌患者入院时LEVT的发生率及其相关危险因素进行了探究，研究发现在入院前均未行手术治疗的肺癌患者LEVT的发病率为5.2%（12/231），而在77例肺良性疾病患者入院时确没有查到LEVT，说明其LEVT的发病率在这些良性疾病中是非常低的，从而提示肺癌患者LEVT的发生率在入院时明显高于肺良性疾病患者。这个结果也与文献报道的恶性肿瘤血栓事件的发病率明显高于良性疾病是相符的。恶性肿瘤血栓事件的发病率与肿瘤自身因素有密切相关，其中，恶性肿瘤细胞可产生组织因子（tissue factor, TF）、癌性促凝物质（cancer procoagulant, CP）、细胞因子和炎症因子等可直接从凝血酶原和血小板多个途径激活凝血，从而使患者处于高凝状态^[15, 16]^。

术前肺癌患者发生LEVT相关危险因素的分析中发现，入院时伴有远处转移的肺癌患者更易发生LEVT。相关研究亦报道，晚期肿瘤是VTE发生的独立高危因素。如在对34, 000多例癌症患者的研究分析中发现，伴有VTE发生的癌症患者更有可能处于疾病的晚期^[17, 18]^。有研究^[4]^认为接受化疗的癌症患者每年VTE的发病率估计为11%，这一风险可能会上升到20%或更高；同时，化疗使癌症患者VTE的复发风险增加了2倍。本中心19例化疗患者中有2例存在LEDVT，虽然发生率与上述相似，但是与非化疗者无统计学差异。这可能与本中心收治化疗患者样本量不足和患者化疗次数少有关。同时在临床上许多化疗患者是术后辅助化疗，这部分患者的VTE事件是由化疗引起，还是由手术引起（手术后新发，一直存在到术后辅助化疗时），值得进一步研究。

此外，我们的数据提示，入院时伴有LEDVT的肺癌患者，D-Dimer明显高于无LEDVT的肺癌患者；而FIB、PT、APTT、TT和血小板在这两组之间却无统计学差异。D-Dimer是高凝和纤溶活化的主要标志之一^[19, 20]^。在临床上，D-Dimer检查虽已经成为筛查VTE的首要检查之一，其敏感度接近99%，但其特异性仅为50%左右。因为，约90%的肺癌患者凝血功能指标异常，表现为血液高凝状态^[21]^。同时本中心数据提示VTE发生范围越大，D-Dimer值可能就越高。因此对D-Dimer值较高的肺癌患者应考虑到VTE事件。

我们中心肺癌患者入院时LEDVT总体发生率约为5%，明显高于肺良性疾病患者。入院时伴有远处转移（包括N3淋巴结转移）的肺癌患者更易发生LEDVT，其中，D-Dimer值较高的患者应考虑到VTE事件发生的可能性。
